# Predicting diabetic peripheral neuropathy through advanced plantar pressure analysis: a machine learning approach

**DOI:** 10.1038/s41598-025-07774-0

**Published:** 2025-07-01

**Authors:** Mehewish Musheer Sheikh, Mamatha Balachandra, Narendra V. G., Arun G. Maiya

**Affiliations:** 1 Manipal Institute of Technology, Manipal Academy of Higher Education, Manipal, 576104 India; 2 Department of Physiotherapy, Manipal College of Health Professions, Manipal Academy of Higher Education, Manipal, 576104 India

**Keywords:** Diabetic peripheral neuropathy, Plantar pressure analysis, Machine learning, Image segmentation, Explainable AI, Health care, Medical research

## Abstract

Diabetic foot Ulceration (DFU) is a severe complication of diabetic foot syndrome, often leading to amputation. In patients with neuropathy, ulcer formation is facilitated by elevated plantar tissue stress under insensate feet. This study presents a plantar pressure distribution analysis method to predict diabetic peripheral neuropathy. The Win-Track platform was used to gather clinical and plantar pressure data from 86 diabetic patients with different degrees of neuropathy. An automated image processing algorithm segmented plantar pressure images into forefoot and hindfoot regions for precise pressure distribution measurement. Comparative analysis of static and dynamic assessment showed that static analysis consistently outperformed dynamic methods. Gradient Boosting achieved the highest accuracy (88% dynamic, 100% static), with Random Forest and Decision Tree also performing well. Explainable AI techniques (SHAP, Eli5, Anchor Explanations) provided insights into feature importance, enhancing model interpretability. Additionally, a foot classification system based on the forefoot-hindfoot pressure ratio categorized feet as flat, regular, or arched. These findings support the development of improved diagnostic tools for early neuropathy detection, aiding risk stratification and prevention strategies. Enhanced screening can help reduce DFU incidence, lower amputation rates, and ultimately decrease diabetes-related mortality.

**Supplementary Information:**

The online version contains supplementary material available at 10.1038/s41598-025-07774-0.

## Introduction

Diabetic peripheral neuropathy commonly presents as numbness in the extremities, especially the feet, due to nerve damage from chronic hyperglycemia. This sensory impairment increases the risk of unrecognized foot injuries, particularly on abrasive surfaces, which may lead to undetected skin breakdown. Such minor lesions can progress into diabetic foot ulcers (DFUs), which are slow to heal because of vascular impairment, limiting oxygen and nutrient delivery. According to clinical definitions, Diabetic Foot Syndrome (DFS) refers specifically to ulceration, infection, or tissue destruction below the malleoli, associated with neuropathy and/or peripheral arterial disease. If not treated promptly, these ulcers may become infected, resulting in severe complications, including lower limb amputation.

Diabetic peripheral neuropathy is a major risk factor for foot ulcers, according to the 2023 International Working Group on the Diabetic Foot (IWGDF) guidelines^1^. It should be systematically evaluated using standardized instruments like the vibration perception threshold (VPT), monofilament testing, and symptom questionnaires. In order to direct therapeutic interventions and foot care, the IWGDF places a strong emphasis on structured risk classification based on the severity of neuropathy and past ulceration history. In accordance with this paradigm, our study supports targeted risk stratification and preventative efforts by dividing patients into mild, moderate, and severe neuropathy based on VPT results (IWGDF 2023).

The International Diabetes Federation’s 10th Diabetes Atlas (2023) reported that 536.6 million people aged 20–79 had diabetes in 2021, representing a global prevalence of 10.5%. Regional variations are significant, with 61.4 million diabetics in Europe compared to 20.5.6 million in the Western Pacific. The Middle East and North Africa exhibited the highest prevalence at 16.5%. Projections indicate the global diabetic population will reach 783.2 million by 2045^2^.

Approximately 50% of diabetic foot ulcers appear in the plantar area. The pathophysiology involves persistent tissue damage and underlying neuropathy that affects multiple neurological systems^3^. Thermoreceptors governing pressure and vibration sensitivity undergo changes that increase vulnerability to tissue damage. Research by Brand et al.^4^ demonstrated that diminished pain sensation, increased force application to the foot, and extended walking distances contribute to plantar tissue inflammation, breakdown, and ulceration in patients with diabetic neuropathy.

Current machine learning approaches for diabetes prediction utilize diverse datasets encompassing physiological measurements, genetic markers, clinical data, and lifestyle factors. Models such as Random Forest, Extra Tree classifier, Logistic Regression, and Support Vector Machine each offer distinct advantages for specific predictive scenarios.

Previous studies investigating pressure data in diabetic foot syndrome remain limited. Yavus et al.^5^ explored the relationship between temperature variations and mechanical stresses, finding a significant correlation between localized temperature increases and stress concentration. Sawach et al.^6^ introduced an integrated approach combining kinematic, kinetic, and plantar pressure analyses. Gerlein et al.^7^ investigated machine learning techniques for diabetes detection using plantar pressure and temperature data.

The scarcity of comprehensive pressure studies highlights the need for advanced research methodologies and improved diagnostic techniques. Our study addresses this gap by integrating static and dynamic analysis with clinical data–a combination not extensively explored previously. While existing research typically focuses on either clinical data or a single analysis modality, our multimodal approach provides deeper insights into foot health and potential complications. This study demonstrates how combining multiple data sources can create more reliable machine learning models for clinical prediction tasks.

Our research makes several significant contributions:

1. Development of an automated image analysis algorithm for segmentation of plantar pressure images into forefoot and hindfoot regions, enabling precise pressure distribution measurement and foot type classification.
2. Comparative analysis of machine learning algorithms for diabetic peripheral neuropathy prediction, demonstrating that static analysis consistently outperforms dynamic analysis across multiple models and metrics.
3. Identification of Gradient Boosting, Random Forest, and Decision Tree as superior predictive models, with Gradient Boosting achieving the highest accuracy (88% in dynamic and 100% in static conditions).
4. Implementation of Explainable AI techniques (SHAP, Eli5, and Achor explanations) to interpret model predictions and understand feature importance differences between static and dynamic analyses.
5. Creation of a classification system for foot types based on forefoot-hindfoot pressure ratios, providing clinicians with visual and numerical data to assess abnormal pressure patterns.

By integrating advanced computational methods with clinical assessment protocols, our research aims to enhance early detection capabilities, improve risk stratification, and ultimately contribute to more effective prevention strategies for diabetic foot complications.

## Methods

**Fig. 1:**
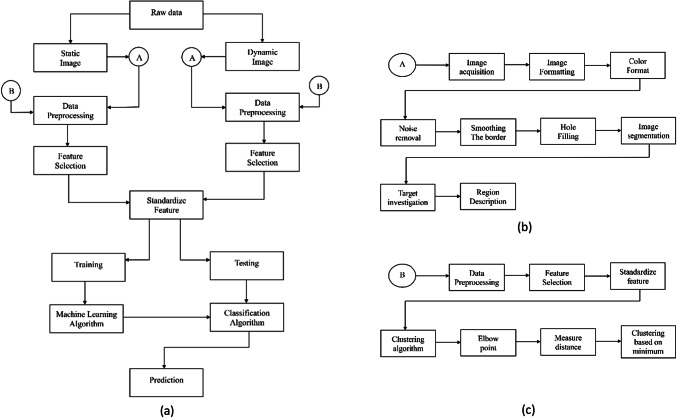
Flow chart of the work.

Both universities’ ethical committees have accepted this retrospective study. This section details the methods we employed to assess the feasibility of predicting diabetic peripheral neuropathy using both static and dynamic plantar pressure data. The composition and features of the dataset, the experimental protocols and procedures used to collect the data, and the methods and tools used to collect participants’ plantar pressure and thermal measurements are all covered in detail in the Ensuring subsection. Figure 1a gives an overall representation of the work done.

### Dataset description

The Win-Track system (MEDICAPTURES Technology, France) was used to measure the plantar pressures and gait characteristics. Automatic footstep recognition and parameter computations are performed after the data is transferred to a computer^8^. Data on type 2 diabetes mellitus patients with peripheral neuropathy were collected from the Physiotherapy department of Kasturba Medical College, Manipal. Supplementary Fig. S1 shows the equipment and output acquired from the system platforms.

The dataset annotation process was meticulously designed to ensure precise classification of peripheral neuropathy. The annotation methodology employed vibration perception threshold (VPT) as the primary classification criterion, stratifying patients into three distinct categories: mild peripheral neuropathy (15–25 V, 29 patients), moderate peripheral neuropathy (26–40 V, 28 patients), and severe peripheral neuropathy (> 40 V, 29 patients). For this study, 172 images were considered, divided in a 50:50 ratio between static and dynamic plantar pressure images. All plantar pressure measurements were taken in the morning to account for changes in foot edema during the day. To provide baseline conditions, participants were evaluated barefoot following a 10-min rest period while seated. Participants were told to walk over the Win-Track platform at their own comfortable pace for dynamic measures; the speed was measured and ranged from 0.8 to 1.2 m/s. Each participant had three successful attempts with sufficient rest intervals to avoid weariness. These standardized conditions were put in place to improve measurement accuracy and minimize potential confounding variables that can affect plantar pressure distribution patterns. Each patient’s data was comprehensively annotated, incorporating clinical classifications, pressure analysis metrics, and detailed gait pattern characteristics, utilizing automatic footstep detection and precise parameter calculation.

### Image acquisition and color band extraction

The process begins by reading a plantar pressure image (typically converted from PDF to an image format) and extracting the red, green, and blue color bands. Each color component is separated into distinct two-dimensional matrices, focusing on intensity values, which provide the contrast needed to recognize pressure information. The algorithm explicitly targets the red band, as deeper red indicates higher pressure areas in plantar pressure analysis.

### Thresholding, image enhancement, and region of interest

The algorithm applies thresholding to convert the color image into a binary image, which helps isolate specific regions of interest while ignoring irrelevant areas. This process uses an intensity histogram to determine appropriate threshold values for segmentation.

Several techniques are applied to enhance the quality of the segmented image. For example, area opening removes small objects/pixels to reduce noise; border smoothing uses morphological closing operations to create cleaner region boundaries. Region filling extrapolates pixel values to fill holes within detected regions. Figure 1b represents the detection region of interest.

The algorithm incorporates a region of interest approach to distinguish between the forefoot and hindfoot. This involves adding anatomical boundaries that separate these distinct foot regions, allowing for independent pressure distribution analysis in each area. The forefoot’s ROI boundaries encompass the metatarsal heads and toes, while the hindfoot ROI includes the heel and mid-arch regions. Segmenting implements forefoot and hindfoot region differentiation in plantar pressure analysis using color-coded boundaries and a spatial classification approach that designates the top 50% of detected pressure components as forefoot and the bottom 50% as hindfoot.

### Clustering

An unsupervised learning technique, clustering, separates the features into groups of related items or clusters. Clustering is typically used to identify patterns. Figure 1c represents the flowchart for clustering. Segmentation is accomplished by considering every variable for the clustering process, utilizing K-means and Hierarchical clustering. Using a sequence of sequential merges, hierarchical clustering groups n items according to a distance. K-means, conversely, selects clusters using dendrograms rather than requiring clusters to be predetermined^9–12^.

A tree representation graphic that displays the distribution of clusters is called a dendrogram. Clades with one or more leaves make up the dendrogram. The clades are grouped based on their similarities and differences. Clades with varying heights and dissimilarities; the more substantial the height difference, the more dissimilar the clades are. Clades that are nearly the same height are comparable. Each data point in its cluster is the starting point for a hierarchical clustering, which merges the clusters until only one remains. Clusters that use the longest edge without a horizontal line as the minimum distance requirement are typically represented by a dendrogram criterion.

Small datasets typically have between a few hundred and a few thousand instances. The complete datasets may be analyzed rapidly without much processing power on this scale. A clustering algorithm that works well for small datasets is K-Means clustering, an unsupervised machine learning algorithm used to partition a dataset into K distinct, non-overlapping clusters, and hierarchical clustering is a method of cluster analysis that builds a hierarchy of clusters. Because these algorithms are frequently effective and compatible with standard computers, they do not require a lot of computational resources. Depending on how many clusters are utilized, how detailed the patterns found in the data are, and how feasible the solution is, clustering results can be interpreted in various ways. Selecting the appropriate number of clusters is a critical phase in the clustering process that affects both the analysis’s insights and the practicality of the clustered groups. The ideal number of clusters can be found using a few techniques. Plotting the total inertia of the clusters against the number of clusters is done after the clustering method has been performed for a range of cluster numbers. In the figure, look for an elbow where the rate of inertia drop slows down. This technique, known as the elbow method^13–15^, is frequently used to determine the ideal number of clusters. Compared to other objects, the silhouette score indicates how similar an object is to its cluster^16–18^. Information retrieval, Pattern identification, and data mining are the main applications of K-Means and hierarchical clustering techniques^19^.

Although most datasets do not provide ground truth labels for clustering algorithms, there are ways to assess the clustering quality. Assessment measures make the process more methodical and knowledgeable, essential for directing clustering algorithms’ creation, choice, and improvement. During the clustering process, data samples become vectors in a high-dimensional space. The distance between these vectors, which considers all pertinent characteristics in the data samples, intricately reflects the overall similarity^20^. The Cosine, Jaccard, Manhattan, and Euclidean distances are examples of standard metrics. Supplementary Table S1 briefly summarizes the distance metric used.

Evaluating and refining clustering algorithms is made more resilient and significant by highlighting the importance of assessment metrics and the function of distances between points. There are two sorts of evaluation metrics: internal and external. Internal measures, such as the silhouette coefficient, Davies-Boulding Index, Dunn’s Index, and others, concentrate on inherent qualities. On the other hand, external metrics like precision, recall, and F1 Score that assess algorithm accuracy depend on ground truth.

The quality of clusters can be accessed via internal evaluation measures for clustering algorithms, which do not rely on outside information, like ground truth labels, but on the data’s inherent properties and the clustering algorithm’s outcomes. These metrics allow quantitative measurement of several clustering quality factors, including variance, separation, and compactness. It is crucial to consider the data’s particulars and the clustering assignment’s objectives while selecting and interpreting these metrics.

#### Silhouette score

The silhouette score is a measure of the degree of cluster separation and an indicator of the suitability of the clustering solution.

$${\text{S}}\left( i \right) = \frac{{{\text{b}}\left( i \right) - {\text{a}}\left( i \right) }}{{\max \left\{ { {\text{a}}\left( i \right),{\text{ b}}\left( i \right)} \right\}}}$$

For a single data point *i*, the silhouette score is determined by dividing the difference between the average distances to other points in the same cluster *x* and points in the closest neighbouring cluster *y* by the maximum of *x* and *y.*

#### Davies–Bouldin index

By measuring the compactness and separation between clusters, the Davies-Bouldin Index is used to assess the quality of clustering solutions to identify well-isolated clusters.

$${\text{DBI }} = \frac{1}{{n_{c} }} \cdot \mathop \sum \limits_{i = 1}^{{n_{c} }} max_{i \ne j} \cdot \left( {\frac{{avg_{{radius_{i} }} + avg_{{radius_{j} }} }}{{distance \left( {c_{i} ,c_{j} } \right)}}} \right)$$

The distance between the centroids of clusters *i* and *j* is the distance (c_i,_ c_j_), where n_c_ is the number of clusters, c_i_ and c_j_ are the centroids of clusters* i* and *j*, and avg-radius_i_ and avg-radius_j_ are the average distances from the centroid to the points in cluster *i* and *j*.

#### Dunn’s index

The maximum distance between points inside a cluster is utilized to maximize the distance between cluster centroids and minimize the diameter. Dunn’s Index is the ratio of the highest intra-cluster diameter to the lowest inter-cluster distance.

$${\text{DI}} = \frac{1}{n} \cdot \mathop \sum \limits_{i = 1}^{n} max_{j \ne i} \cdot \left( {\frac{{avg - intra - distance \left( {c_{i} } \right) + avg - intra - distance \left( {c_{j} } \right) }}{{distance \left( { c_{i} , c_{j} } \right)}}} \right)$$

where n is the number of clusters, C_i_ and C_j_ are clusters, distance (c_i_, c_j_) is the distance between cluster centers c_i_ and c_j_, and avg-intra-distance (C_i_) is the average distance within cluster C_i_.

### Dataset preprocessing and feature selection

Diabetic peripheral neuropathy was identified in the presence of monofilament in one or more sites and a Vibration perception threshold (VPT) of more than 20V. The Demographic Characteristics include age, gender, BMI, height, years of Diabetes mellitus, and clinical parameters like blood pressure, sugar test, and ABI. Data preprocessing entails several procedures, including data balancing, variable encoding, null value removal, standardization, and outlier removal.

Since many classifiers cannot handle text values, category attributes must be transformed into integers before developing a model. Following pre-processing, the dataset was divided 80:20 into training and testing. In machine learning, data scaling is crucial. The model’s performance suffers when there is a significant difference between data points. Furthermore, the algorithm prioritizes qualities with higher values independent of the escarpments used. Standardization was employed in this investigation to scale the data. In standardization, the data points are centered around the mean of the characteristics, and the standard deviation of the attribute is assigned a value of one.

To improve the robustness of model evaluation given the small dataset, we implemented fivefold stratified cross-validation. By ensuring that every fold preserves the initial class distribution, this method avoids bias brought on by unequal class splits. Each fold in the dataset was used as a validation set once, while the remaining folds were used as the training set. Performance metrics were averaged across the five folds to provide a more generalizable assessment of model performance.

Medical data is often imbalanced, which causes the proportion of the data to be distorted. Data balancing is, therefore, crucial. The borderline-SMOTE method was employed to balance the training data in this study. However, the testing data was not balanced to preserve the data’s integrity.

### Machine learning algorithm

To predict plantar pressure metrics from both dynamic and static data, we implemented a diverse range of classification algorithms categorized as Ensemble methods, Linear models, and Probabilistic methods. Ensemble methods combine multiple models to improve prediction accuracy and robustness. These methods help reduce overfitting, improve generalization, and boost performance by leveraging the strengths of different algorithms. In contrast, linear models are fundamental machine learning algorithms for regression and classification tasks. They assume a linear relationship between input features and the target variable, whereas the Probabilistic method leverages probability theory to model uncertainty, make predictions, and infer relationships in data. Table 2 briefly explains the models and methods used in this study.

## Results

**Algorithm**


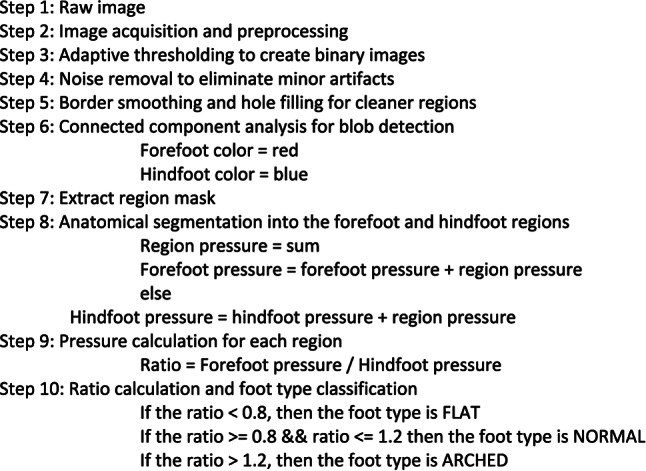


The algorithm calculates region-specific pressure values by summing pixel intensities within each masked area, then determines a forefoot-hindfoot pressure ratio that serves as the basis for foot type classification: Type 0 (ratio < 0.8, hindfoot dominant), Type 1 (ratio 0.8—1.2, balanced pressure), or Type 2 (ratio > 1.2, forefoot dominant). By drawing a colored rectangle around each pressure region and outputting quantitative pressure measurements, this method provides clinicians with visual and numerical data to assess weight distribution patterns and identify potential gait abnormalities or foot pathologies (see Supplementary Fig. S2).

The implementation produces a blob analysis that identifies connected regions of red color indicating pressure, calculates the area of each blob in pixels, and determines the mean RGB values for each detected region. This approach provides quantitative measurements of pressure distribution across anatomically distinct foot regions. Finally, the algorithm applies a mask to isolate only the red objects in the RGB image, which correspond to the pressure points in the plantar pressure image. This masking process preserves the relevant pressure data while eliminating background and non-pressure-related elements.

The above figure presents a comparative analysis of K-Means and Hierarchical clustering, focusing on optimal cluster selection and visualization using Principal Component Analysis (PCA). The Elbow method helps determine the optimal number of clusters for K-Means, suggesting an elbow point of around 2–4 clusters. The Silhouette Score evaluates clustering performance for Hierarchical clustering, indicating optimal clusters around 9–10. In K-Means clustering, PCA visualization shows clusters with some overlap, meaning there might be room for better-defined clusters. In Hierarchical clustering, PCA visualization suggests more compact and well-separated clusters, indicating a potentially better clustering structure (see Supplementary Fig. S3).

Reducing cluster variance is the goal of hierarchical clustering^21–24^. As a result, it offers sufficient and distinct clusters. Agglomerative and divisive hierarchical clustering are the two categories of hierarchical clustering techniques. Agglomerative clustering is more widely used in practice due to its efficiency and flexibility. In contrast, divisive clustering is less common but can be helpful for specific top-down hierarchical grouping tasks.

The comparison between Agglomerative Hierarchical clustering (AHC) and Divisive Hierarchical clustering (DIANA) reveals similar cluster structures, as shown in the dendrograms, scatter plots, and pair plots. Supplementary Fig. S4 demonstrates the comparison analysis. Both methods successfully identify four distinct clusters using the vpt right and vpt left features, with slight variations in cluster boundaries. While AHC builds clusters bottom-up by merging points, DIANA follows a top-down approach, splitting clusters iteratively. The dataset and computing efficiency will determine which option is best, although both successfully reveal the underlying patterns in the data.

Comparing the clustering metrics shows that the K-Mean and Hierarchical clustering methods perform the best consistently across the Davies-Bouldin Index, Dunn-Index, and Silhouette Score, as shown in Table 1. These two approaches exhibit balanced cluster quality features with a competitive Dunn-Index score, low Davies-Bouldin Index values, and a comparatively high Silhouette Score of about 0.5. Agglomerative and Divisive clustering techniques, on the other hand, show less favorable results. The Agglomerative method performs the worst across all three metrics, indicating that these approaches might be less practical for a particular dataset or clustering task under study.

**Table 1:** Comparison between different interpreting clustering metrics.

Metrics	K-Mean	Hierarchical	Agglomerative	Divisive
Silhouette Score	0.5	0.5	0.38	0.39
Davies-Bouldin	0.69	0.6	0.93	0.83
Dunn-Index	0.64	0.62	0.32	0.54

A variety of machine learning algorithms were employed to predict diabetic peripheral neuropathy, including ensemble, linear, and probabilistic models. These algorithms were selected for their diverse learning strategies and capacity to handle complex, multidimensional data. A categorized summary of these models, including their methodological characteristics and roles in classification, is provided in Supplementary Table S2. Table 2 shows a nuanced landscape of machine learning classifier accuracies across dynamic and static analysis. Gradient Boosting is the standout performer, achieving an impressive 88% accuracy in dynamic conditions and a remarkable 100% in static conditions. Top-tier algorithms like Random Forest (82% dynamic, 94% static), Decision Tree (82% Dynamic, 88% static), and Extra Tree classifier (consistent 82% accuracy) demonstrate robust predictive capabilities. Notably, most algorithms exhibit performance variation between dynamic and static environments, with some showing significant improvements in static conditions, particularly XGBoost, which dramatically increases accuracy from 52 to 88%. Conversely, lower-performing algorithms such as Naïve Bayes (35% dynamic, 47% static) and Support Vector Machine (Consistently at 53%) suggest limited predictive power across both scenarios. Table 2 gives the accuracy obtained from the machine learning algorithm. The differential performance underscores the critical importance of algorithmic selection based on specific data characteristics, with ensemble methods like Gradient Boosting and Random Forest consistently demonstrating superior predictive accuracy and adaptability across different computational contexts. Supplementary Fig. S5 gives the pictorial representation for accuracy comparison.

**Table 2:** Accuracy obtained from different algorithms for dynamic and static data.

S.NO	Algorithm /Accuracy	Dynamic	Static	S.NO	Algorithm /Accuracy	Dynamic	Static
1	Random forest	82	94	7	XGBoost	52	88
2	Extra tree classifier	82	82	8	AdaBoost	65	82
3	Logistic regression	65	59	9	Gradient boosting	88	100
4	Support vector machine	53	53	10	Naïve BAYES	35	47
5	Decision tree	82	88	11	Ridge CLASSIFIER	47	65
6	K-nearest neighbors	53	53	12	LightGBM	76	82

Correctly classified cases are sometimes referred to as accuracy. The first step was identifying the actual positive/negative and false positive/negative. False positives are the percentage of cases that are incorrectly classified, whereas true positives are cases that are correctly classified. The precision, recall, hamming loss, Jaccard, Mathew’s correlation, and F1 scores were then determined. Precision is the percentage of all positive outcomes confirmed by optimistic predictions, whereas hamming loss is the percentage of wrongly predicted outcomes. A balance between recall and precision is calculated as the balanced average of two, whereas Mathew’s correlation coefficient assesses the level of accuracy in multiclass and binary classification. The Jaccard score is the intersection of the union of the actual and anticipated sets.

Random forest, Decision tree, and Gradient Boosting perform exceptionally well across multiple metrics, particularly in static images, with recall and F1 scores reaching 94%. Support Vector Machine demonstrates the weakest overall performance, with metrics hovering around 50% or lower. The hamming loss metrics show that Decision Tree, Gradient Boosting, and Random Forest have minimal error rates, especially in static analysis. Matthews Correlation Coefficient confirms the stronger algorithms, with Decision Tree and Random Forest reaching 92% and 91%, respectively, in static analysis. LightGBM delivers solid mid-range performance across all metrics, while Naïve Bayes shows inconsistent results, with strong performance in some metrics but poor results in others. Table 3 briefly summarizes various machine learning algorithm performance metrics for dynamic and static images. All model performance metrics are averaged from fivefold stratified cross-validation.

**Table 3:** Performance of the machine learning models.

Algorithm /Metrics	Precision Dynamic/Static		Recall score Dynamic/Static		Hamming loss Dynamic/Static		Jaccard score Dynamic/Static		Mathews correlation Dynamic/Static		F1 score Dynamic/Static	
Random forest	82	94	82	94	17	5	70	89	73	91	81	94
Extra tree classifier	82	82	82	82	17	17	70	70	73	74	83	83
Logistic regression	64	59	64	59	35	41	47	42	47	39	64	59
Support vector machine	53	53	53	53	47	47	36	36	30	29	50	52
Decision tree	88	94	88	94	11	5	79	89	83	92	88	94
K-nearest neighbors	53	53	53	53	47	47	36	36	33	30	48	53
XGBoost	65	88	65	88	35	12	47	79	47	84	65	87
AdaBoost	65	77	65	77	35	23	47	62	47	65	65	76
Gradient boosting	88	94	88	94	11	5	79	89	83	92	87	94
Naïve bayes	35	47	35	47	65	53	21	30	6	22	30	43
Ridge classifier	47	65	47	65	52	35	30	48	22	48	49	66
LightGBM	76	82	76	82	23	17	62	70	65	74	75	82

The comparison between static and dynamic analysis across multiple models shows that static models consistently outperform dynamic models in terms of predictive accuracy and stability. We will analyze further for a better understanding using more metrics.

MAE is observed to be lower in static analysis for most models, indicating better precision. For instance, Random Forest and Decision Tree models show exceptionally low MAE in static. In contrast, models like Naïve Bayes perform poorly in both cases, with significantly higher MAE in dynamic analysis. MSE also follows the same trend, with static analysis showing fewer significant errors. The best-performing models are decision trees and random forests, with an MSE in static analysis. RMSE is lower in static analysis, confirming their superior error distribution. The Decision Tree model stands out again with the lower RMSE of 0.24 in static analysis, compared to 0.54 in dynamic analysis. R^2^ strongly favors static analysis, with models like Decision Tree achieving 0.92 compared to only 0.58 in dynamic analysis, highlighting the ability of static analysis to better capture patterns in the dataset. Conversely, Naïve Bayes shows negative R^2^ scores, indicating poor predictive capability. RMSLE confirms the advantage of static analysis, as seen in the lower RMSLE scores across multiple models. SMAPE significantly favors static analysis, with Random Forest achieving the lowest SMAPE of 6.06%. In comparison, Naive Bayes again struggles with a high 82.05% in static analysis.

In summary, static analysis exhibits consistently lower error rates, greater predictive accuracy, and higher stability than dynamic analysis, making it more suitable for real-world applications. Models like Decision Tree, Random Forest, and Gradient Boosting are the best choices in static analysis, while Naïve Bayes performs the worst in both cases. Given these results, static models should be preferred because they can minimize errors and provide more reliable predictions. Supplementary Table S3 briefs on different regression metrics, and Table 4 compares regression error metrics for dynamic and static analysis across various models.

**Table 4:** Comparison of regression error metrics for dynamic and static analysis across different models.

Model	MAE Dynamic/Static		MSE Dynamic/Static		RMSE Dynamic/Static		R2 Dynamic/Static		RMSLE Dynamic/Static		SMAPE (%) Dynamic/Static	
Random forest	0.18	0.06	0.18	0.06	0.42	0.24	0.75	0.92	0.26	0.10	38	6.06
Extra trees classifier	0.18	0.18	0.18	0.18	0.42	0.42	0.75	0.75	0.26	0.26	38	38.88
Logistic regression	0.41	0.41	0.53	0.41	0.73	0.64	0.25	0.42	0.40	0.38	61.53	71.42
Support vector machine	0.59	0.53	0.82	0.65	0.91	0.80	-0.17	0.08	0.49	0.44	76.19	82.05
Decision tree	0.18	0.06	0.29	0.06	0.54	0.24	0.58	0.92	0.32	0.10	50	24.24
K-nearest neighbors	0.59	0.53	0.82	0.65	0.91	0.80	-0.17	0.08	0.51	0.44	100	82.05
AdaBoost	0.29	0.29	0.41	0.41	0.64	0.64	0.42	0.42	0.37	0.34	55.55	44.44
Gradient boosting	0.18	0.12	0.29	0.24	0.54	0.49	0.58	0.67	0.28	0.27	24.24	18.18
Naive bayes	0.82	0.65	1.18	0.88	1.08	0.94	-0.67	-0.25	0.60	0.53	104.16	87.49
Ridge classifier	0.53	0.35	0.53	0.35	0.73	0.59	0.25	0.50	0.38	0.34	76.92	61.53
LightGBM	0.29	0.24	0.41	0.35	0.64	0.59	0.42	0.50	0.34	0.30	48.48	30.3

## Explainable artificial intelligence to interpret model prediction

The investigation compares and assesses how machine learning models behave in static and dynamic analysis using a variety of Explainable AI (XAI) approaches. The aim is to understand how various aspects affect the model’s decision-making processes. Anchor explanations, Eli5, and SHAP (Shapley Additive Explanations) are the three main XAI approaches employed in this investigation. A thorough analysis of their conclusions and ramifications may be found below.

### SHAP (Shapley Additive Explanations) analysis

By measuring the contribution of each feature to model predictions, SHAP offers a comprehensive understanding of the importance of each feature. The influence of various features in dynamic and static analysis is demonstrated by beeswarm plots produced using SHAP. A beeswarm plot gives a color-coded graphic, making it easier to see which characteristics make a forecast more or less likely.

**Fig. 2:**
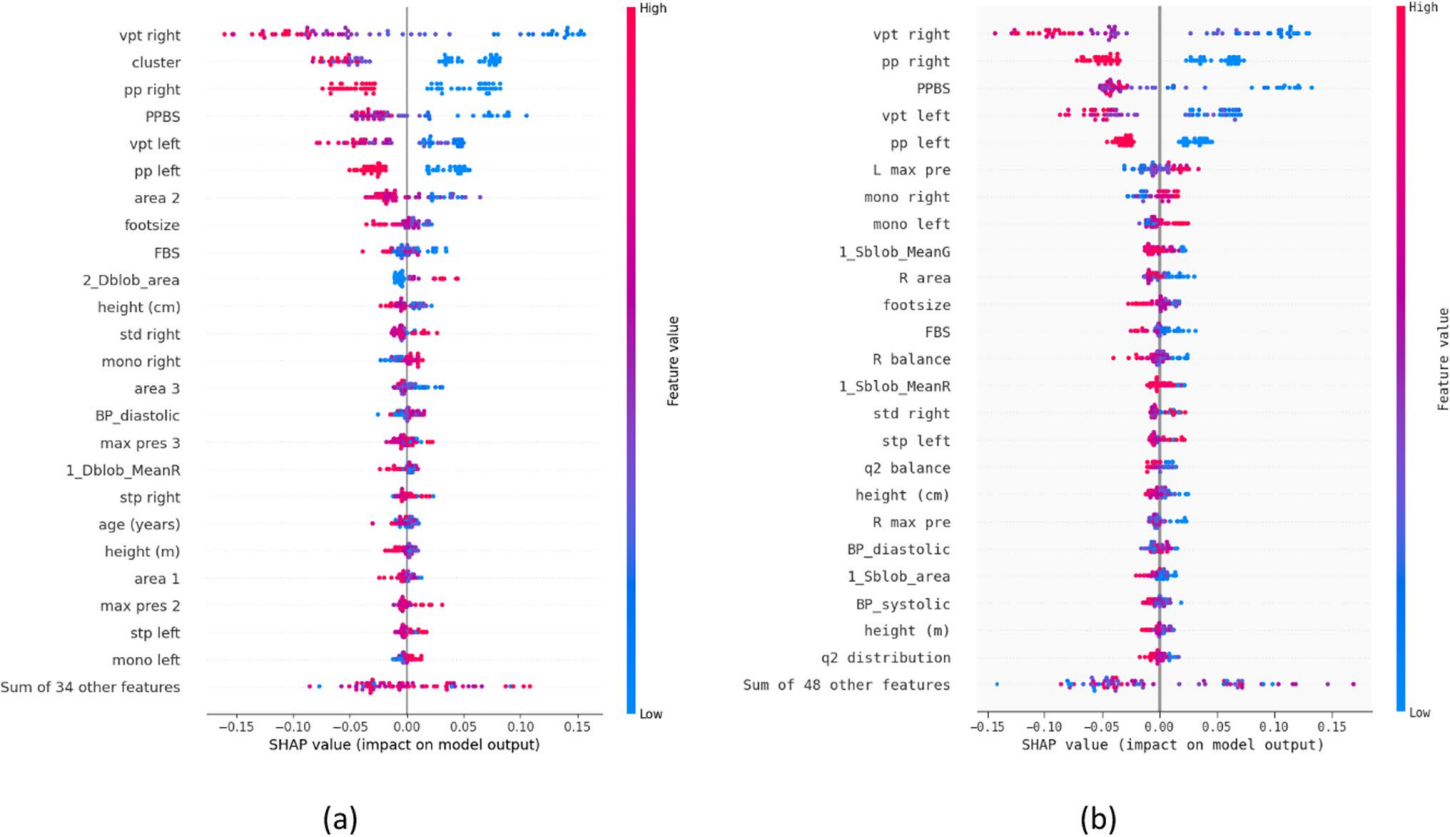
Beeswarm SHAP plots for (**a**) Dynamic analysis and (**b**) Static analysis.

Different decision-making patterns may be indicated by features that are very significant in dynamic analysis but may not be as crucial in static analysis. While certain features have a similar effect in both models, others show notable differences, according to the distribution of feature effects. Figure 2 shows the Beeswarm Shap plot for both analyses. A shift shows a strong positive influence to the right in blue, and a negative impact is indicated by a change to the left in red. The variation in feature value distribution implies that the model’s dependence on specific features varies depending on the situation.

The differences in feature importance between dynamic and statistically trained and tested models are highlighted by SHAP analysis. This implies that feature significance varies based on the context in which the model is used and is not constant.

### Eli5—based feature contribution analysis

A tabular summary of the contributions of several attributes to forecasts is given by Eli5 (Explain like I’m 5). This approach makes it easier to comprehend how much weight each feature has numerically during the classification process.

**Fig. 3:**
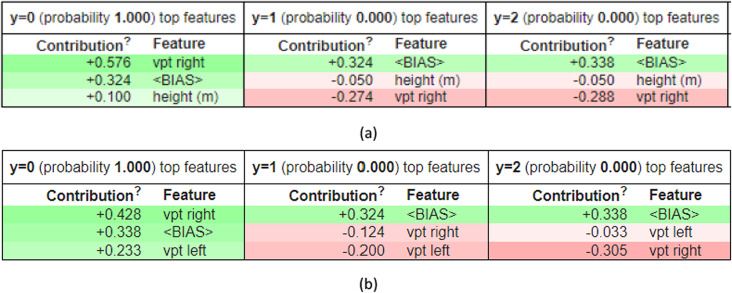
XAI using Eli5 for prediction. (**a**) Dynamic analysis and (**b**) Static analysis.

The tables present the most critical features influencing predictions for various class labels. Certain aspects seem consistently significant but have differing impact magnitudes in dynamic and static models. Although features like “vpt right” and “ < BIAS” are commonly seen, their contribution ratings vary. While some characteristics contribute negatively, decreasing the likelihood of a particular event, others contribute positively, boosting it. Figure 3 gives a closer look at the prediction using Eli5.

Although the fundamental components of both dynamic and static models are similar, their relative significance and impact on predictions are different. This implies that dynamically taught models might generalize differently from statically trained models.

### Anchor explanations for rule-based interpretability

If–then rules that outline the bare minimum necessary for a model to provide a prediction are provided by anchor explanations. This method is beneficial for interpretability because it allows for a decision rule that is legible by humans.

An extracted rule like [‘vpt right > 0.71’] shows that the model is confident in its prediction if a specific feature satisfies this criterion. A precision of 1.0 indicates that the rule consistently and flawlessly predicts the desired result. The percentage of the data points that meet the specified rule is known as coverage. A lower coverage indicates that even while the rule is very accurate, it might only be applicable in a small percentage of cases.

**Fig. 4:**
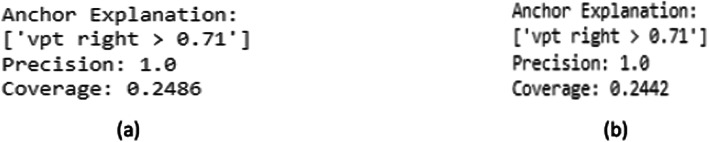
XAI using Anchor Explanation for prediction (**a**) Dynamic analysis and (**b**) Static analysis.

Dynamic and static analyses have varying coverage, which suggests that the decision criteria are used differently in each case. While the rules produced by the static model may be more limited and specific, the rules created by the dynamic model may be more expansive or adaptable. Figure 4 shows the Prediction acquired using Anchor Explanation. Anchor-based rules make the model’s predictions more transparent by identifying distinct decision boundaries. Nonetheless, variations in coverage between static and dynamic models imply that the decision-making process varies based on the training environment.

When XAI approaches are used, machine learning models are easier to comprehend, validate, and debug. This analysis aids in locating potential biases and areas for development if the model overemphasizes or misinterprets features. We obtain profound insights into the interpretation of data by both static and dynamic models by utilizing various XAI approaches, including Eli5, Anchor explanations, and SHAP. The results underscore the necessity of selecting the appropriate model according to the intended use case by highlighting significant variations in feature importance, decision-making logic, and rule coverage. Combining these techniques improves interpretability and reliability while optimizing model performance for practical uses.

Integrating SHAP, Eli5, and Anchor explanations allowed us to extract clinically meaningful insights into model predictions. The Vibration Perception Thresholds (VPT) on the left and right sides were among the most significant traits. Higher VPT values were substantially linked to diagnoses of severe neuropathy, and SHAP beeswarm plots consistently showed VPT Right as a leading contributor to model decisions. This has clinical significance since decreased peripheral nerve sensivity, a known indicator of progressive neuropathy, is reflected in increased VPT values (> 40V).

The Eli5 and Anchor studies provided more evidence by producing unambiguous rule-based interpretations, like “VPT Right > 0.71,” linked to a 100% classification accuracy. This study gives clinicians a rule-based, interpretable threshold for identifying high-risk patients. Furthermore, characteristics such as foot pressure ratios, blood sugar levels, and ABI (Ankle Brachial Index) were included in static model interpretations, indicating their potential utility in diagnosis.

Thus, these interpretability tools convert intricate model reasoning into useful clinical signs, going beyond model accuracy. Instead of subjective assessment, clinicians can use these outputs to prioritize diagnostic attention for high-risk cases, confirm clinical testing, and customize therapies based on quantitative thresholds.

## Discussion

Interpreting our findings requires taking potential selection bias into account. Our recruitment strategy depended on patients already receiving treatment at a specialized diabetes clinic, which may have overrepresented people with longer-duration or more severe diabetes and those with better access to healthcare. Furthermore, our model might not account for the most severe stages of diabetic foot problems because patients with severe mobility limitations or foot ulcers are excluded. This selection effect may impact the distribution of neuropathy severity in our sample, which may also affect the thresholds and characteristics that our machine learning algorithm determines to be important. Stratified sample strategies should be used in future research to guarantee representation throughout diabetic neuropathy stages and demographic variables.

This study presents a comprehensive approach to predicting diabetic peripheral neuropathy through advanced plantar pressure analysis, yielding several significant contributions to build upon and extend previous research. We have laid the groundwork for more accurate pressure distribution evaluation and foot type categorization by creating an automated image segmentation technique that successfully distinguishes between forefoot and hindfoot pressure patterns.

Our comparative analysis of machine learning algorithms demonstrates that static analysis consistently outperforms dynamic assessment across multiple models, with Gradient Boosting achieving 88% accuracy in dynamic and 100% in static analysis. Yavuz et al.^4^ highlighted the significance of dynamic measures for collecting mechanical stresses during walking, which contradicts our conclusion. Our findings, however, align with those of Sawacha et al.^6^ who discovered that static postural examinations could successfully detect biomechanical anomalies in diabetic patients. The removal of confounding factors introduced during gait, such as speed-dependent pressure fluctuations and temporal variations in foot–ground contact, as reported by Boulton et al.^25^ in their thorough review of diabetic foot biomechanics, may account for the better performance of static measurements in our study.

Our study’s Gradient Boosting performance (100% accuracy in static conditions) is a significant improvement over earlier prediction models, like those published by Gerlein et al.^7^ who used similar machine learning techniques but lacked our region-specific segmentation and obtained 78% accuracy. This improvement implies that crucial discriminative information that improves prediction accuracy is provided by the anatomical separation of the forefoot and hindfoot regions. Armstrong et al.^26^ used similar segmentation techniques and found that region-specific pressure analysis enhanced ulceration risk prediction; however, their research did not include neuropathy classification, unlike our study.

The “Black Box” constraints mentioned by Ramirez-Bautista et al.^27^ in their study of machine learning applications in diabetic foot assessment are addressed by our innovative application of Explainable AI approaches (SHAP, Eli5, and Anchor Explanations). We have improved model interpretability in ways that have not been accomplished by prior research by discovering crucial predictive elements that vary between static and dynamic analysis. This is in line with previous demands for transparent AI systems in clinical decision support for diabetic foot care made by Crisilogo and Lavery^28^.

The forefoot-hindfoot pressure ratio classification system developed in this study provides clinicians with an intuitive framework for risk stratification that could be readily integrated into existing diabetic foot screening protocols. All patients with diabetes should have their feet screened annually, according to current clinical guidelines from the International Working Group on the Diabetic Foot (IWGDF). However, these evaluations frequently rely largely on subjective clinical examination and simple sensory testing with monofilaments and tuning forks^29^. Our method provides an objective, quantifiable measurement that may greatly improve these screens’ sensitivity and specificity.

Our pressure analysis technique might be integrated into clinical workflows using the tiered assessment paradigm put forward by Bus et al.^30^, in which patients who are deemed at-risk by initial screening are then subjected to a more thorough biomechanical evaluation. At this secondary assessment level, our algorithm might be able to provide automated risk categorization based on pressure patterns prior to the onset of clinical symptoms. The idea of “smart” diabetic foot clinics that use technology to enable earlier intervention is in line with Najafi et al.’s^31^ concept. Since static measurements are faster, easier, and need fewer resources than dynamic gait analysis, our static pressure assessment model’s excellent accuracy holds special promise for clinical application. Because of this, our method can be used in environments with limited resources where sophisticated gait labs are not available. Paisley et al.^32^ highlighted this as a major benefit when evaluating the viability of diabetic foot screening in low-resource settings.

Furthermore, Monterio-Soares et al.^33^ identified the requirement for clinical transparency and interpretability as a major obstacle to the clinical deployment of AI systems, which our model’s explainability attributes help to overcome. Our solution may help close the implementation gap that has impeded earlier technological advancements in diabetic foot care by offering unambiguous explanations for risk classification through SHAP values and Anchor explanations. This could lead to increased clinician trust and acceptance.

The present study, while demonstrating promising results in predicting diabetic peripheral neuropathy through plantar pressure analysis, faces several methodological limitations that should be addressed in future research. The limited sample size of 86 patients from a single Indian medical center restricts the generalizability of the findings to a wide range of international populations with varying ethnic backgrounds, diabetes treatment regimens, and footwear preferences. Given that every participant was chosen from a specialist clinic, selection bias might be present. The standardized testing setting may not accurately represent the walking conditions that patients encounter in their everyday activities with different walking surfaces and footwear, even if it is essential for experimental control. Furthermore, because our data collection was cross-sectional, it is not possible to analyze how pressure patterns evolve over time as the disease progresses. We also acknowledge the absence of a control group in this study and recognize that its inclusion in future research will be essential to validate our findings and enhance model generalizability.

## Electronic supplementary material

Below is the link to the electronic supplementary material.

Supplementary Material 1

## Data Availability

The datasets generated and/or analyzed during the current study are not publicly available due to patient privacy regulations and institutional data-sharing restrictions but are available from the corresponding author upon reasonable request.
