# Supplementary material for: Predicting diabetic peripheral neuropathy through advanced plantar pressure analysis: a machine learning approach
Source: Sci Rep. 2025 Jul 1;15:20962. doi: 10.1038/s41598-025-07774-0 (PMC12215299; doi:10.1038/s41598-025-07774-0)
Supplement: Supplementary file 1 — Supplementary Material 1 [file 41598_2025_7774_MOESM1_ESM.docx]

| 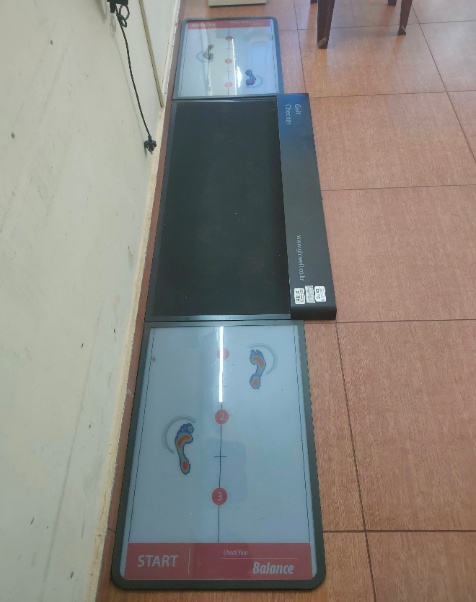  (a) | 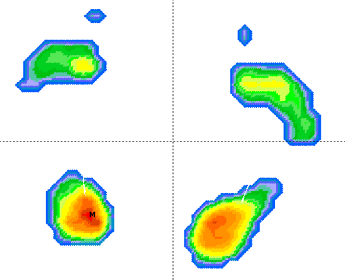  (b) |
| --- | --- |
|  | 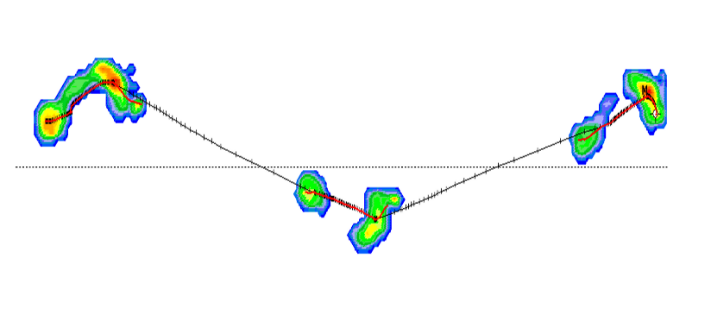  (c) |

**Supplementary Figure S1**: (a) Win-Track platform used to measure the plantar pressures and gait characteristics.

This figure illustrates the plantar pressure distribution across different foot regions in diabetic patients with varying neuropathy severity. High-pressure zones are highlighted, indicating areas at risk for ulceration.

| 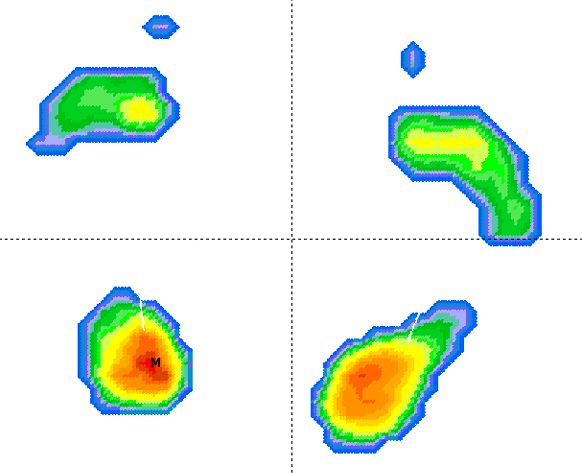  (a) | 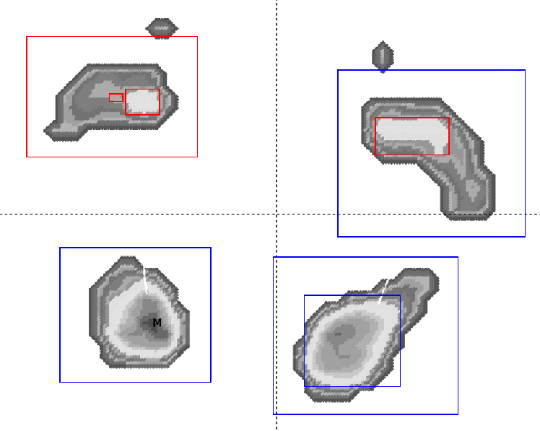  (b) |
| --- | --- |

**Supplementary Figure S2:** Pressure analysis of a (a) Static raw image and (b) Segmented image.

This anatomical segmentation enables more precise clinical assessment by providing region-specific pressure measurements. It facilitates comparative analysis between forefoot and hindfoot loading patterns, which is crucial for identifying gait abnormalities and pressure-related pathologies.

| 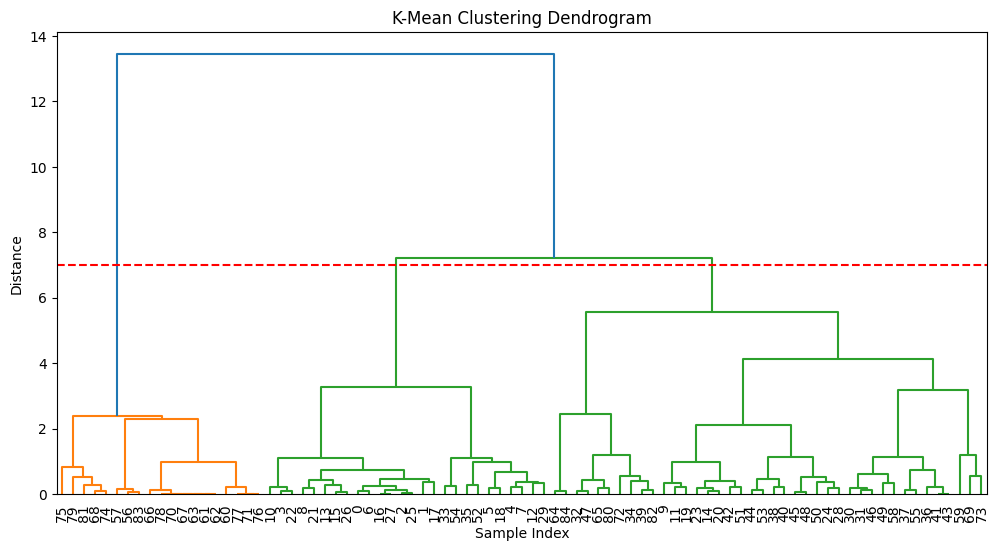 | 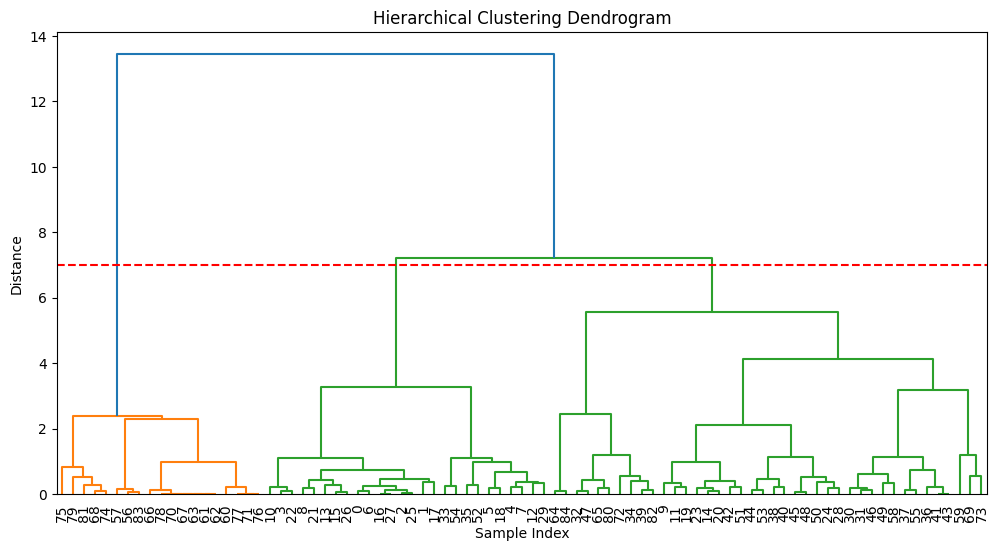 |
| --- | --- |
| 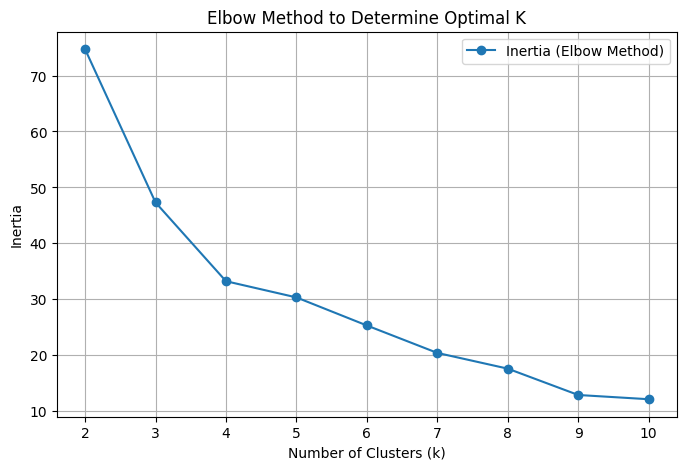 | 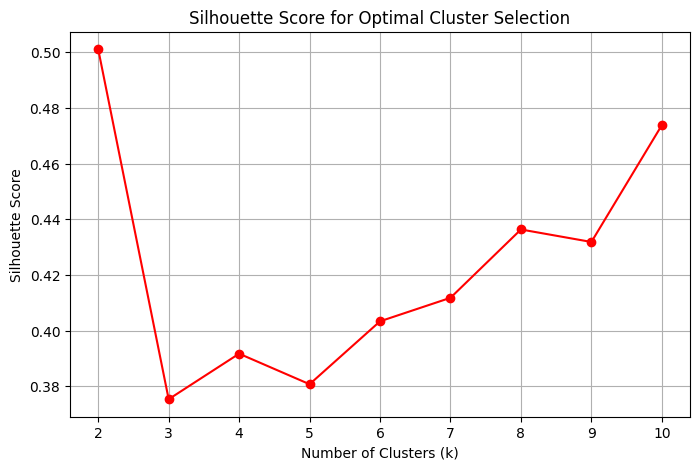 |
| 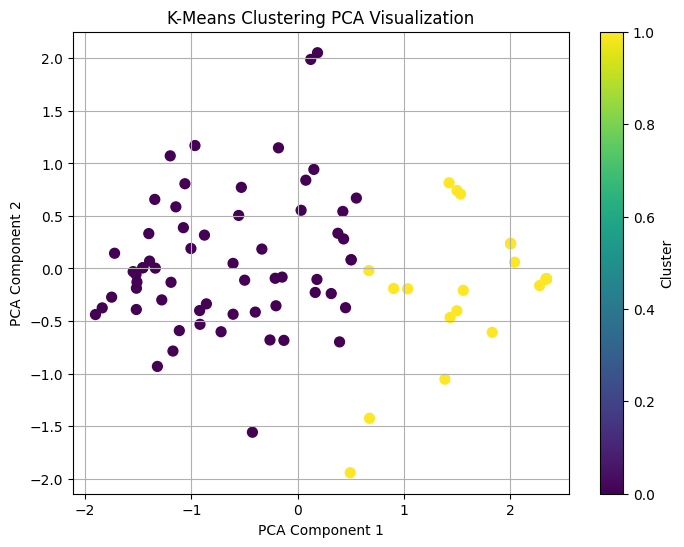 | 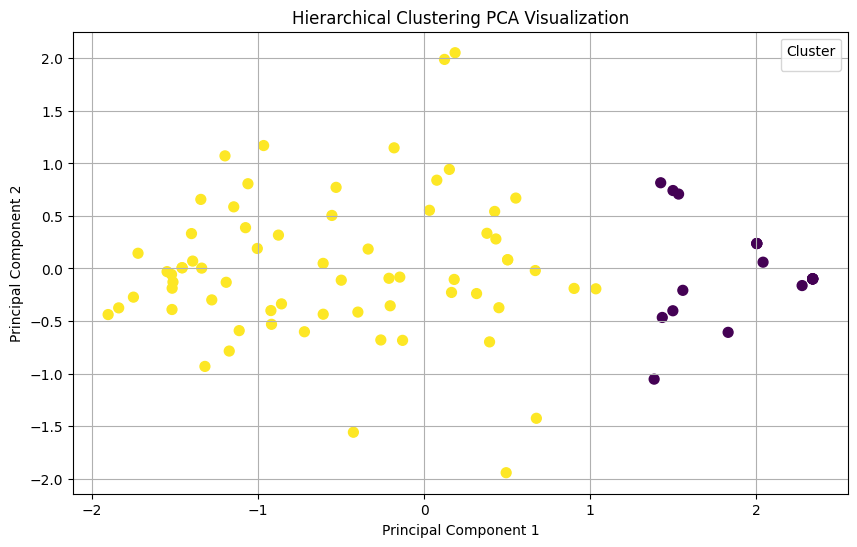 |
| (a) | (b) |

**Supplementary Figure S3: D**etermining the optimal number of clusters and PCA visualization for (a) K-mean and (b) Hierarchical clustering.

The dendrograms for both methods show a similar hierarchical structure, but the approach to forming clusters differs significantly. The PCA visualizations reveal that both methods yield identical cluster distribution, reinforcing that the final clusters are comparable, regardless of whether a bottom-up or top-down approach is used. The density and pair plots further analyze how clusters are distributed across multiple features, indicating that some clusters have distinct feature ranges.

| 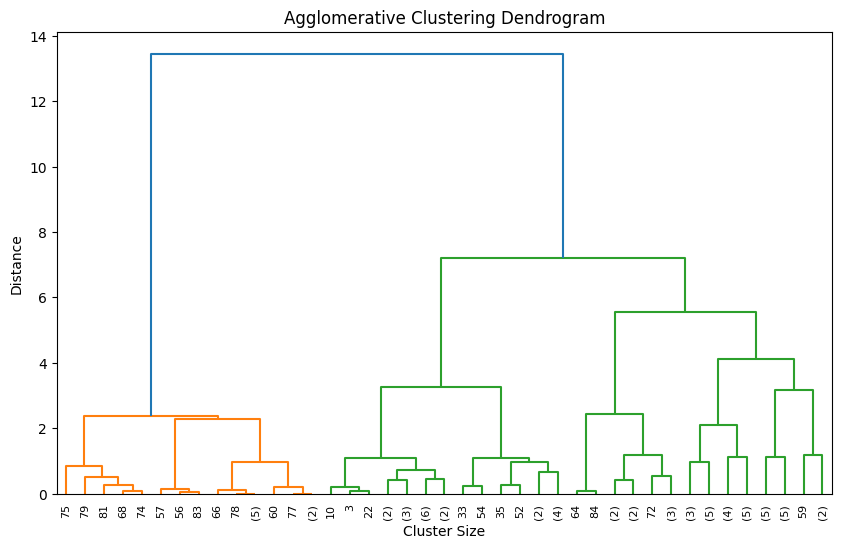 | 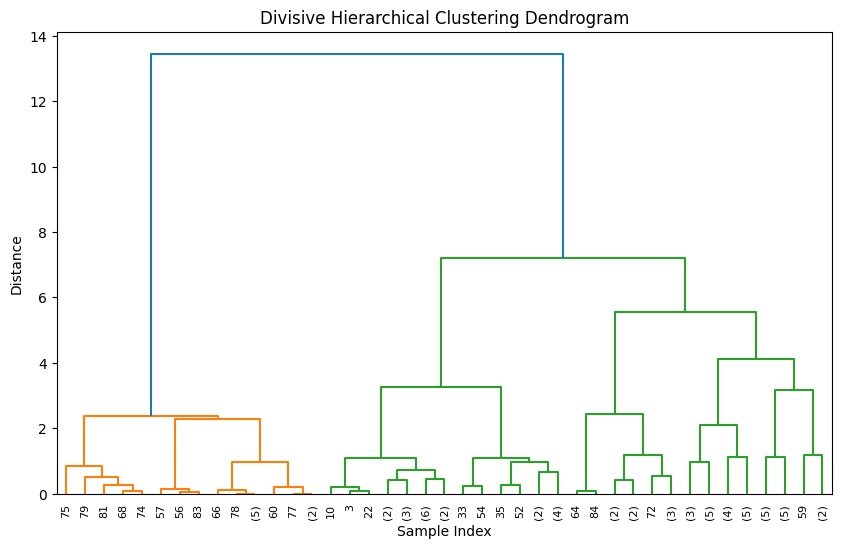 |
| --- | --- |
| 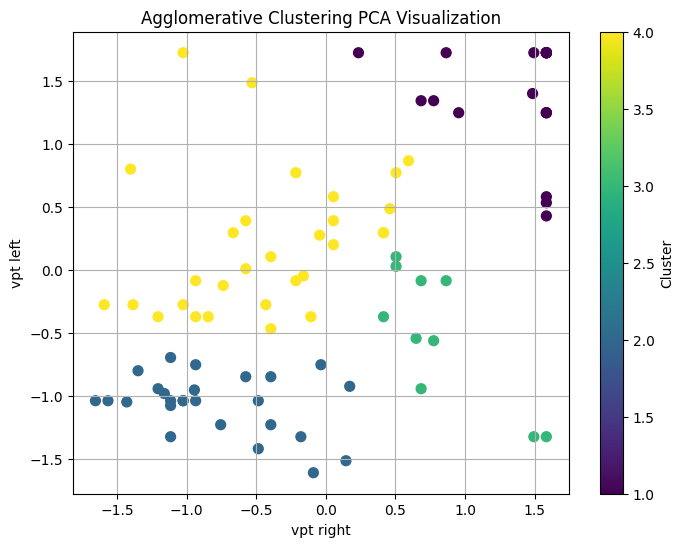 | 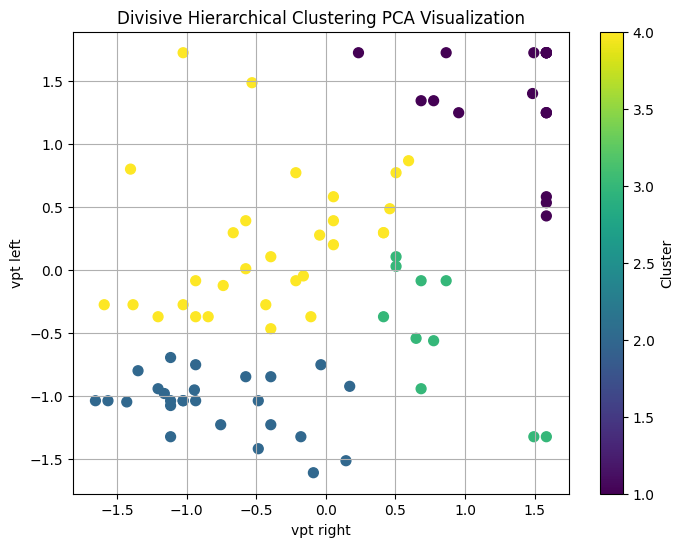 |
| 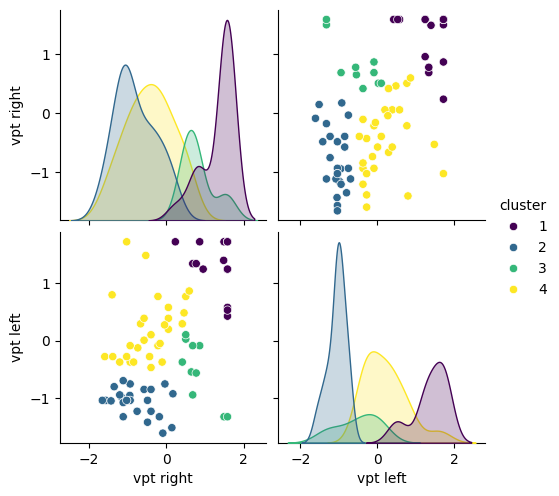 | 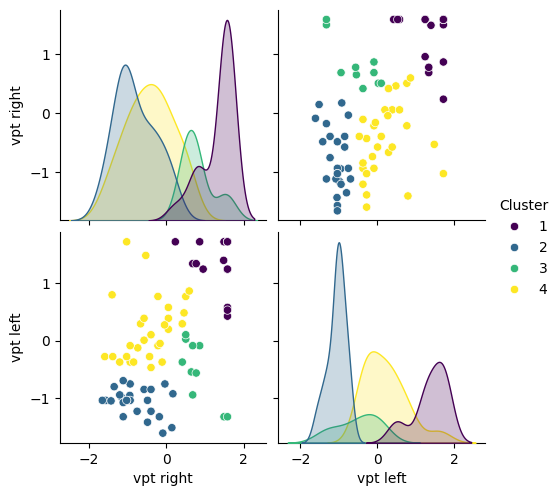 |
| (a) | (b) |

**Supplementary Figure S4**: Clustering results for (a) Agglomerative and (b) Divisive hierarchical clustering.

A more detailed Hierarchical clustering analysis comparing the agglomerative and Divisive approaches. The agglomerative method starts with each point as its cluster and merges them iteratively, forming a tree-like structure from the bottom up. In contrast, the divisive method starts with all points in a single cluster and splits them progressively into smaller groups.

| 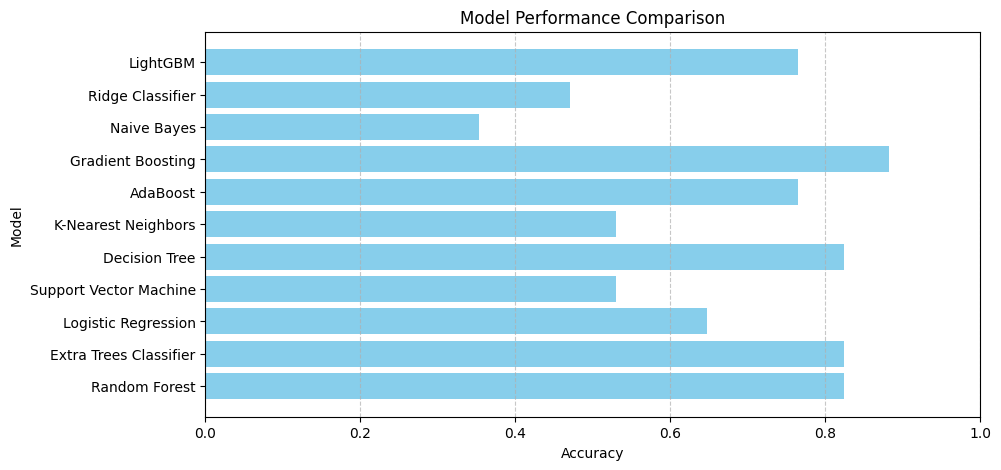  (a) |
| --- |
| 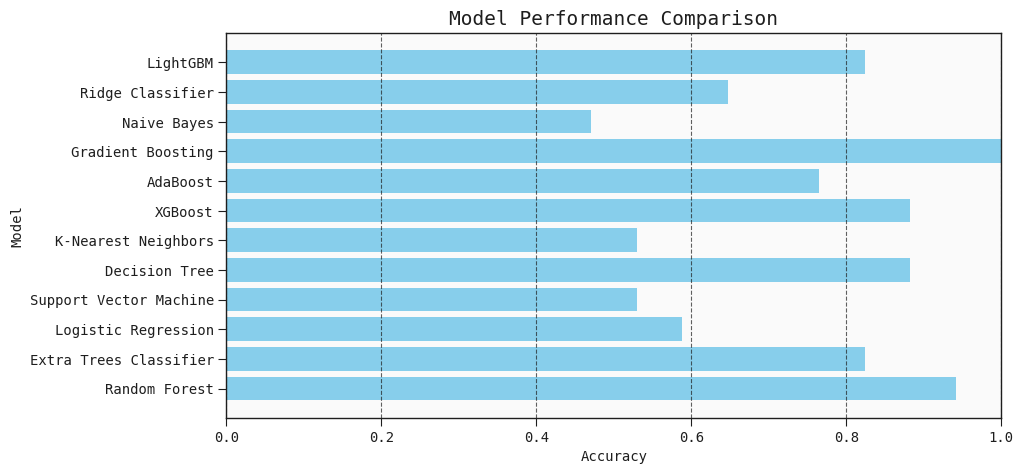  (b) |

**Supplementary Figure S5:** Accuracy model performance comparison for (a) Dynamic and (b) Static analysis

A bar chart comparing the accuracy of different machine learning models, including Gradient Boosting, Random Forest, Support Vector Machine, and others.

**Supplementary Table S1:** Standard distance metrics

| Metric | Definition | Formula | Sensitivity |
| --- | --- | --- | --- |
| Cosine | Measures the angle between two vectors in high-dimensional space. | *similarity*(*A*,*B*)= $\frac{A.B}{\left\vert\left\vert A \right\vert\right\vert\left\vert\left\vert B \right\vert\right\vert}$  where A and B are angles between two data points | Low |
| Euclidean | Measures the shortest straight-line distance between two points. | *d*(*p*,*q*)$=\sqrt{\sum_{i=1}^{n} {(p_{i}- q_{i})}^{2}}$​ where p and q are two data points, and n is the number of dimensions | High |
| Jaccard | Measures dissimilarity between sample sets. | *J*(*A*,*B*)=$\frac{\mid A\cup B\mid}{\mid A\cap B\mid}$  where A and B are the number of features shared by two data points ​ | Moderate |
| Manhattan | Measures the sum of absolute differences in each dimension. | *d*(*p*,*q*)=$\sum_{i-1}^{n} \vert p_{i}- q_{i}\vert$  where p and q are two data points, and n is the number of dimensions | High |

This table presents different distance metrics used in machine learning and data analysis, including Cosine, Euclidean, Jaccard, and Manhattan distance, along with their formulas and sensitivity levels.

**Supplementary Table S2:** Regression models

| **Methods / Models** | **Category** | **Definition** |
| --- | --- | --- |
| AdaBoost | Ensemble | Adaptively weights classifiers and training instances to improve classification accuracy. |
| Extra Tree Classifier | Ensemble | Employs increased randomization in feature selection and threshold determination compared to Random Forest. |
| Gradient Boost | Ensemble | Sequentially builds weak classifiers into a strong predictive model by focusing on previously misclassified instances. |
| Light GBM | Ensemble | Offers efficient memory usage and handles large datasets through leaf-wise tree growth. |
| Logistic regression | Linear | Model’s binary outcome probabilities using a logistic function. |
| Naïve Bayes | Probabilistic | Applies Bayes’ theorem with the conditional independence assumption between features. |
| Random Forest | Ensemble | Aggregate predictions from multiple randomized decision trees to reduce variance and enhance generalization. |
| Ridge Classifier | Linear | Incorporates L2 regularization to address multicollinearity issues. |
| Support Vector Machine | Linear | Identifies optimal hyperplanes to maximize the margin between classes. |

Summary of various machine learning models categorized as ensemble, probabilistic, or linear, briefly describing their functionality. Classifier performance was compared across multiple statistical metrics, including Precision, Recall, F1 Score, Jaccard Score, Hamming Loss, and Matthews Correlation Coefficient (MCC).

**Supplementary Table S3:** Briefing Regression metrics

| **Metric** | **Definition** | **Sensitivity** | **Interpretability** |
| --- | --- | --- | --- |
| Mean Absolute Error (MAE) | Measures the average absolute difference between prediction and actual values | Low | High |
| Mean Squared Error (MSE), | Measure of the average squared errors | High | Moderate |
| Root Mean Squared Error (RMSE), | Measure the square root of MSE | High | High |
| R-squared (R^2^ ) | Measures how well a model explains the variance in data | Low | High |
| Root Mean Squared Log Error (RMSLE), | Measure the relative difference in predicted and actual values | Low | Low |
| Symmetric Mean Absolute Percentage Error (SMAPE) | Percentage-based metrics are crucial for understanding prediction accuracy relative to the actual values | Moderate | Moderate |

This table lists key evaluation metrics for regression models, such as Mean Absolute Error (MAE), Root Mean Squared Error (RMSE), and R-squared (R^2^), along with their sensitivity and interpretability levels.
